# Modeling and assessing Highly Pathogenic Avian Influenza (HPAI) spread, epidemiological control measures, and cost

**DOI:** 10.1371/journal.pone.0340004

**Published:** 2026-04-30

**Authors:** Eihab Fathelrahman, Magdi Mohamed Ali, Tamrat Gebiso Challa, Raeda Osman, Adil I. Elawad, Mervat H. AL Nuaimat, Meera M. Saeed, Yassir M. Eltahir, Oum-Keltoum Bensalah, Youssef El-Khatib, Aaron Reeves

**Affiliations:** 1 Department of Integrative Agriculture (INAG), College of Agriculture & Veterinary Medicine (CAVM), United Arab Emirates University (UAEU), Al Ain, United Arab Emirates; 2 Biosecurity Section, Ministry of Climate Change and Environment (MoCCAE), Dubai, United Arab Emirates; 3 Animal Development & Health, Ministry of Climate Change & Environment (MOCCAE), Dubai, United Arab Emirates; 4 Animal Extension and Health Services Division, Abu Dhabi Agricultural and Food Safety Authority (ADAFSA), Abu Dhabi, United Arab Emirates; 5 Department of Mathematical Sciences, College of Science, United Arab Emirates University (UAEU), Al Ain, United Arab Emirates; 6 Centre for Public Health Surveillance and Technology, RTI International, Research Triangle Park, Raleigh, North Carolina, United States of America; Central Laboratory for Evaluation of Veterinary Biologics, Agricultural Research Center, EGYP

## Abstract

Highly Pathogenic Avian Influenza (HPAI) is a highly contagious, transboundary disease that can affect animals and humans, posing significant health risks to both. This study aimed to prepare for a potential outbreak by modelling the spread of HPAI using stochastic methods with the customized North American Animal Disease Spread Model (NAADSM) software ^©^, which was tailored specifically for the UAE. Information from 46 commercial poultry farms, including parents, broilers, and layers, was used. This investigation simulated one baseline scenario without intervention and three disease control methods. The scenarios are baseline (without any disease-spread intervention; scenario 1), movement control strategy (scenario 2), intensive vaccination strategy (scenario 3), and depopulation of poultry farms (scenario 4). The model generated outputs on the number of farms and the number of birds infected, vaccinated, or depopulated, as well as the duration of the disease outbreak. The results indicated that the depopulation strategy, scenario 4, led to the lowest epidemic impact and the shortest outbreak duration (16 days), compared with scenarios 1, 2, and 3, which had outbreak durations of 26, 32, and 31 days, respectively. The results of this study help decision-makers determine the optimal control strategy to combat HPAI. Such a strategy minimizes the consequences of the disease spread and the associated implementation costs.

## Introduction

Serious health hazards to both humans and animals are posed by Highly Pathogenic Avian Influenza (HPAI), a highly infectious transboundary disease recognized as zoonotic. It results in significant mortality rates among both domestic and wild birds [1,2]. HPAI causes the loss of poultry production, including a shortage of meat and egg supplies shortage [3,4]. Recent HPAI outbreaks led to catastrophic effects, moving outside of Asia to Europe, Africa, America, and the rest of the world [5–7]. It has become a challenge to poultry production globally due to its high transmissibility and fatality [8]. Avian Influenza (AI) virus was first detected as Low Pathogenic Avian Influenza (LPAI) in 1959 and continued as a rare disease until it appeared as an outbreak between 1998 and 2003 [7,9]. However, the H5 haemagglutinin of the A/Goose/Guangdong/1/1996 (Gs/Gd) lineage continued to significantly affect the rest of the world since its first detection in 1996 [10–12]. Since then, the H5 subtype strain of HPAI has dominated the outbreaks worldwide in countries such as the Netherlands, Asian countries, and the United States [13]. The virus has continued to circulate as LPAI in some regions until it caused a major epidemic wave in China in 2013 [14]. This wave persisted for five consecutive years, after which the virus mutated into an HPAI strain around May 2016, acquiring a multi-basic cleavage site in the HA gene. The resulting HPAI H7N9 strain caused severe disease in poultry and increased virulence in humans, with confirmed human cases reported from late 2016 onward [15].

Avian influenza A viruses are classified into two groups, LPAI and HPAI, based on their potential to cause disease in chickens [16,17]. The majority of avian influenza A virus cases are low-pathogenic, meaning they can infect domestic birds while causing little to no symptoms in wild birds [18]. That means, they cause either no disease or only moderate illness [19]. Most of the time, they cause ruffled feathers and reduced egg production. However, some of the LPAI viruses can mutate into HPAI viruses in poultry. On the contrary, HPAI viruses cause severe disease and mortality rates of up to 90% to 100% in infected poultry within 48 hours [20]. Practically, only some avian influenza A(H5) and A(H7) viruses are categorized under HPAI A viruses.

Although genetic and antigenic differences exist between influenza A viruses that infect only birds and those that infect birds and people, HPAI and LPAI viruses can spread rapidly in poultry flocks [21]. The A-type HPAI is widespread in the animal kingdom, primarily occurring in birds, humans, horses, pigs, and occasionally in cetaceans and mustelids [10,22]. There is a tremendous growing concern about the viral infection, which is causing substantial annual morbidity and mortality worldwide, particularly for infants, the elderly, and the immunocompromised [10,23]. The virus has undergone spontaneous mutations, including an antigenic shift (A/H5N1) in 1996 in Guangdong, China, in a goose (goose/GD/96 lineage). It has evolved into the 2024 H5N1 clade 2.3.4.4b, which has acquired a transformed ability to transmit to mammals.

LPAI virus infects respiratory and enteric epithelial cells, whereas HPAI infects nearly all organs and tissues; this systemic infection pattern often has fatal outcomes [24]. This is due to differences in the sites of viral replication. Mutated viral particles transform LPIA into HPAI, which infects domestic waterfowl and poultry. Wild migratory birds could reintroduce them, potentially disseminating the infection over long distances and causing outbreaks in specific regions [25]. Ducks primarily sustain influenza A virus transmission within the waterbird community. Ahmed et al [26] studied Bangladesh’s spatiotemporal magnitude and direction of Highly Pathogenic Avian Influenza (H5N1) outbreaks. The authors concluded that geostatistical analysis revealed significant latitudinal trends in outbreak progressions, similar to the detected lines of intensity and magnitude.

In general, the importance of HPAI worldwide has increased significantly due to the substantial economic losses it has caused to the poultry industry and the development of zoonotic antigenic strains, such as the H5N1 virus, from which human disease arises [27]. The World Health Organization (WHO) has reported 956 cases of HPAI H5N1 human infection, of which 464 (49%) are fatalities in 23 countries from December 2003 to July 2024 [1]. Consequently, research focuses on HPAI transmission and control in the event of an outbreak to prevent a widespread poultry epidemic. Several studies have been published to provide decision-makers with more effective, modern tools. Nickbakhsh et al. [28] investigated the impact of LPAI viruses on the transmission and consequences of HPAI outbreaks in poultry populations, providing insights that may inform effective management strategies for avian influenza.

To provide insights into handling upcoming outbreaks in poultry populations, Andronico et al [29] simulated and evaluated the efficacy of various control measures during the 2016–2017 HPAI H5N8 outbreak in southwest France. The study by Ssematimba et al [30] predicts when the HPAI virus will likely infect US poultry flocks during the 2022–2024 pandemic. The research output offers recommendations on improving monitoring, biosecurity protocols, and outbreak management strategies within the sector. Such efforts play a crucial role in disease control planning, protecting regions from the potentially severe socioeconomic repercussions of an HPAI outbreak, and decreasing potential human exposure [31].

Few studies have assessed the spread of HPAI as well as evaluated control measures, calculating associated governmental expenses [32,33]. Furthermore, disease spread modeling can be used to analyze the effect of different disease spread control strategies on disease transmission and to estimate the associated government costs in disease control processes [34]. Disease modeling will also help us better understand the existence, transmission, and prevalence of the HPAI virus [32]. Additionally, disease modeling plays a crucial role in predicting disease risk and developing intervention and control plans that help slow the development of the disease and improve readiness for the spread of HPAI at chicken farms [35]. The important information generated by this research can be utilized by policymakers, development professionals, and poultry farm owners to prevent infections and prepare for ongoing, early disease management and eradication efforts.

In numerous low- and middle-income nations, the implementation of depopulation or mass culling of poultry to manage highly pathogenic avian influenza (HPAI) has been challenging to execute effectively [36]. Logistical delays, inadequate or postponed compensation, and insufficient collaboration from farmers frequently hinder prompt responses, while underreporting and secretive poultry movements often worsen the virus’s spread [37]. Insights from Asia and Africa indicate that even with stringent depopulation efforts, HPAI has established itself as endemic in various areas, highlighting the shortcomings of relying solely on culling as a viable control approach [38].

Vaccination has been implemented as a complementary or alternative strategy in several developing nations, including Egypt, Vietnam, and Indonesia. Nonetheless, its effectiveness has been limited by fundamental issues, including insufficient funding, ineffective monitoring and evaluation frameworks, inadequate training for veterinary staff, and poor biosecurity measures during vaccination initiatives [39,40]. Moreover, structural limitations, such as smallholder poultry systems, limited coverage, and the possibility that vaccinated flocks can silently shed the virus, can diminish the sustained effectiveness of vaccination initiatives in resource-limited environments [41]. The identified challenges underscore the need for cohesive control strategies for HPAI that incorporate vaccination alongside enhanced surveillance, active farmer involvement, and robust institutional support.

Poultry production in the United Arab Emirates (UAE) is a growing sector that boosts local supply, improves protein availability, and strengthens food security [42,43]. The UAE’s poultry meat production reached 60,000 metric tons in 2024. This implies that any future HPAI outbreak (if occurred) may have significant economic ramifications to this sector.

Hence, disease control planning is pivotal to protecting the country from the multidimensional impacts of HPAI. The primary objective of disease outbreak modeling is to provide decision-makers with science-based insights to inform and rationalize decisions among available alternative control options. Such information should include (i) the financial and economic impact, as well as (ii) strategies for mitigating it. Numerous studies have shown that this impact can vary across stakeholders, including flocks affected by the disease and unaffected flocks, agribusiness, other sectors of the economy, and consumers [44,45].

### Study objectives

The objective of this study is to model and assess the spread of Highly Pathogenic Avian Influenza (HPAI) in the poultry farms in the UAE and simulate if-then four scenarios of no intervention, movement control, vaccination, and poultry farm depopulation to contain the disease spread to learn about the consequences of such scenarios’ from epidemiology and cost of implementation by the government and recommend the most optimum course of actions.

### Data and methodology

The following sections describe the research data and methodological approach used.

### Data for simulation

The Animal Extension and Health Services Division of the Abu Dhabi Agriculture and Food Safety Authority (ADAFSA) and the Ministry of Climate Change and Environment (MOCCAE) made the data available for this research. Data on poultry was collected from 46 commercial poultry operations across the Emirates of Abu Dhabi, Dubai, Sharjah, and Ras al-Khaimah. The suggested stochastic disease model utilizes a flock of birds in the farm, referred to as a “unit,” as its basis for simulation. The unit data includes the product type, number of birds, geographic location (defined by latitude and longitude), and initial health status (e.g., latent). Production type includes flocks exhibiting similar disease symptoms, detection rates, transmission probabilities, and management strategies. Production type refers to the grouping of bird flocks and the control practices employed for each flock. The identified production types in the UAE, as per the customized poultry farms database, include three categories: broilers, layers, and parent stock units. This model focuses on the commercial sector because, in the country setting, there are no direct or indirect contacts between backyard poultry, live bird markets, and wild birds on the one hand, and the selected large commercial farms on the other hand.

### Methodology

Epidemiologic modeling is a popular technique for simulating and developing different scenarios to predict the potential impacts of infectious disease outbreaks, such as the HPAI virus, on domesticated animal populations [45]. HPAI disease models necessitate three major components. The first component is the poultry population, which encompasses various production types, flock sizes, and geographical locations sensitive to HPAI. The second step involves establishing parameters that describe the disease’s manifestations and transmission. The third and final step involves control techniques, including movement limitations and tracking of direct and indirect connections, which results in increased detection of infected flocks, zone-based surveillance, flock depopulation, and vaccination of at-risk flocks, as assessed by [46]. Several spatially explicit stochastic epidemic simulation models have been developed to evaluate the spread of highly contagious animal diseases and simulate disease outbreaks [47]. In recent decades, stochastic simulation models have been employed to investigate the impacts of contagious animal disease outbreaks and to analyze different control strategies [48,49]. Epidemiologic simulation modeling of HPAI outbreaks offers a valuable framework for estimating their impacts and assessing disease control strategies [13,31].

The North American Animal Disease Spread Model (NAADSM) software was designed to simulate the spread and management of exotic animal diseases in a flock of at-risk livestock [50]. However, the authors customized and adapted the NAADSM framework and software for this study to suit the UAE context. Although different authors employed various classifications of poultry production types [23,31,46], the primary production types in the UAE are Broiler, Layer, and Parent poultry farms. Therefore, this classification was used for HPAI modeling purposes using NAADSM.

To examine the effects of control strategies on rapidly eliminating the HPAI virus, we employed four scenarios in NAADSM software to reduce disease spread and minimize government costs. The initial scenario illustrates the absence of control strategies, indicating a strategy that acknowledges the original circumstances and serves as the basis for the subsequent three methods. The second strategy involves controlling the birds’ movement within the risky areas, starting with a 100% to 10% restriction on movement from day one to day seven. The third scenario represents a strategy based on intensive bird vaccination. In contrast, the fourth scenario represents a strategy of destruction and depopulation, sometimes referred to as “stamping out” the birds in high-risk areas of disease outbreaks.

### Disease states

**Fig 1:**
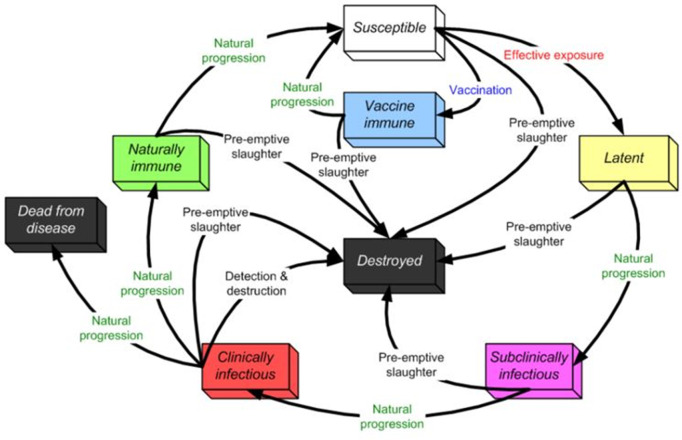
NAADSM simulates states and transitions.

As indicated in Fig 1, this study used NAADSM’s seven discrete disease states. Until a disease control mechanism is implemented, the disease will remain latent once it infects a vulnerable unit within the model. If disease control strategies are not employed, an infected unit will naturally progress from a latent to a subclinical infectious condition. The disease advancement will follow its normal course (see the outer loop of Fig 1). However, as shown in the loop, disease control strategies may interfere with the disease’s natural progression. To run this model, each flock of birds (units) must spend some time in a particular disease state [50].

Units will follow the state progression indicated in the outer loop without intervention. However, intervening actions (i.e., control strategy) may alter the normal disease cycle upon implementing disease control strategies, as illustrated in Fig 1 [50].

### Modelling disease spread in NAADSM

Modeling disease spread parameters in NAADSM considered contact (direct or indirect), airborne, and local area spread methods. A higher likelihood of outbreaks in layer farms was considered, based on previous findings indicating a greater risk of HPAI virus introduction in layer farms than in broiler farms. This elevated risk is attributed to the longer production cycle of layers, which can last up to 18 months [51]. Moreover, the probability of increased contact between farms, particularly through the reuse of cardboard egg trays for egg collection during an epidemic, may further facilitate virus transmission [52,53].

Tables 1 and 2 present the flock-level latent state duration distribution and disease parameters used in the HPAI virus outbreak simulation. As per Longworth et al [54] a one-day latent period was assumed.

**Table 1:** Flock-level latent state duration distribution used in NAADSM.

Parameters description	Production type	Distribution/value	References
**Flock-level latent** **Period duration** **(days)**	All types	Gamma (1.34, 0.18)	[31]
**The probability that infected birds die from the disease**	All types	0.90	[31]

**Table 2:** Flock-level disease parameters used in the simulated HPAI outbreak scenarios.

Natural history of infection (days)^1^		Production types		
		Broilers	Layers	Parents
**Infectious subclinical period**	Minimum	1	1	1
	Maximum	7	16	2
	Most likely	2	6	7
**Infectious clinical period**	Minimum	2	2	2
	Maximum	7	14	7
	Most likely	21	21	21
**Immune** ^2^ **(mimics permanent death from disease)**	(1, 365)	(1, 365)	(1, 365)	(1, 365)

^1^A Probability Distribution Function (PDF) is used in both subclinical and clinical stages.

^2.^The uniform distribution method is used for the immunity period.

### Disease transmission

As shown in Fig 1 Disease transmission between poultry farms was modeled as a function of geographical distance, the probability of airborne and local-region spread per contact, and contact frequency (including both direct and indirect contacts).

### Disease detection

Detection involves identifying and notifying contaminated flocks based on clinical symptoms. Two probabilities influence the overall chance that an infected flock will be detected: the probability of observing clinical signs in a flock given the number of days it exhibited clinical signs, and the probability that the owner or veterinarian will report the disease to animal health authorities. This is based on the number of days since the disease was first detected and reported on any of the birds’ farms.

The delay-to-detection is modeled following Longworth et al. [54] using a BetaPert distribution with the minimum, most likely, and maximum values of 1, 2, and 6 days after the onset of clinical signs. It is assumed that once a farmer detects the first case, if they are familiar with the clinical signs and symptoms of HPAI, they will notify the official authorities. Hence, the probability of detecting the following case will be lower than in the first case and will be modeled using BetaPert distributions with Y = (0, 1, 3).

### Global tracing

When an infected unit is located by tracing one level ahead and backward, NAADSM mimics trace-out investigations by [50]. All contacts, upstream and downstream, are located for each specified farm. The time before tracing takes place follows a Poisson distribution with an average delay of 1 day. Through a similar procedure outlined by Longworth et al [54] Traced farms are subject to the tracing monitoring parameter.

### Disease spread

Although the fecal oral route is the principal transmission pathway of avian influenza among birds, the relative significance of other transmission routes in HPAI remains unclear [55–57]. However, the two main mechanisms to consider are direct bird-to-bird contact (aerosol) and indirect contact via fecal contamination of dust and drinking/feeding equipment (infectious feces), which are most often used in modeling HPAI transmission [58]. Even though additional determinants, such as flock density, exist, the transmission rate is highest for direct contact in flock modelling. In contrast, indirect contact is the most significant transmission mode for HPAI on poultry farms [58]. Direct contact was defined as the movement of birds from one farm to another, such as when they are transferred. The parameters included (poultry birds movement or shipment between units) are: animal shipment mean rate (number of recipient units per source unit per day), distance traveled (probability density function per kilometers), delay in shipping (probability density function per day), the probability of infection of the receiving unit due to exposure to an infected unit and movement rate multiplier (a scalar value based on the number of days since the outbreak was first detected).

In commercial farms using an all-in/all-out production system, one can assume that birds’ direct movements between farms are minimal and that direct contact between farms is almost nonexistent [46,54,58–60]. However, some authors assumed direct bird contact between farms [23,59]. The direct contact frequency was calculated daily using estimates from industry, government, veterinarians, and poultry specialists, based on the production cycle length by production type and the number of barns per farm. Values modeled in NAADSM are taken from [23], with approximately 1:1 replacements of production types, such as ‘parents’, with ‘broiler breeders’, and the average movement distance between each combination of production systems. In the NAADSM, contact frequencies must be specified on a per-day basis. The daily frequency the user enters for each production type pairing specifies the λ parameter for a Poisson distribution [50].

### Transmission probability

HPAI is a highly contagious disease with high transmission efficiency that ranges to 100% probability through direct contact (i.e., movement of birds) from an infected source flock to a non-infected flock [61,62]. Based on the experts’ suggestions and other previous works, the maximum probability is considered in this simulation [23,59]. The probability of the local area spreading the disease transmission mechanism was assumed to be 0.1% within a 3 km radius [54]. Different transmission probabilities were assumed for indirect contact based on the premises distances between premises [23,31,46].

### Indirect contact

Indirect contact encompasses the movement of individuals, such as daily workers, inspectors, and poultry experts, as well as the exchange of supplies and equipment among farms. Indirect contact also includes transportation of poultry products and other items between units. In indirect contact, only sub-clinically infected and clinically infectious units can act as sources of infection, not latent units. The parameters for indirect contact are the estimated daily contact rate and infection transmission probability, as provided in Table 3.

**Table 3:** Indirect contact rate and corresponding HPAI infection transfer probability.

Production type combination	Daily contact rate	Infection transfer probability
**Broiler to Broiler**	0.7513	0.35
**Broiler to Layer**	0.008	0.25
**Broiler to Parent**	0.3653	0.3
**Layer to Broiler**	0.002	0.25
**Layer to layer**	14135	0.35
**Layer to Parent**	0.003	0.25
**Parent to Broiler**	0.4187	0.30
**Parent to Layer**	0.4228	0.25
**Parent to Parent**	1.0698	0.35

Source: Adapted from Lewis et al [23], Patyk et al [31], and Medina et al [46].

### Airborne spread and local spread

Although airborne spread has been hypothesized to play a role in several HPAI outbreaks [63], there is no conclusive evidence. Air sampling within 1 km of infected farms during the 2004 H7N3 HPAI outbreak in British Columbia detected only one sample with low virus levels [64]. Experiments with Newcastle disease have shown that airborne spread occurs only over short distances (<150 m) [65]. This type of spread involves unknown mechanisms (direct or indirect) that act locally to spread infection, including the potential for wild birds to serve as sources of infection and for other sources, such as insects and pests. The authors used a probability of infection of 0.01 at a distance of 1 km from infected premises, decreasing exponentially with increasing distance [23].

### Highly Pathogenic Avian Influenza (HPAI) in UAE control scenarios

This HPAI modeling study considered four scenarios: 1, 2, 3, and 4, representing baseline (no intervention), movement control, vaccination, and depopulation/destruction strategies/control measures, respectively. The first scenario is a control scenario without any intervention, while the second scenario considers controlling poultry farm movements. The third scenario involves intensive vaccination and movement control, and the fourth scenario involves destroying or depopulating infected and susceptible poultry farms. Each scenario is discussed below.

### Baseline/no intervention- Scenario 1

This scenario represents the base scenario of HPAI disease. Under this scenario, three risk zones were examined to show the disease’s spread based on the distance to the infected farm: (i) High (3 km), (ii) Medium (5 km), and (iii) Low (10 km) surveillance zone [66]. For each production type, the possibility of a small outbreak resulting from direct and indirect contact tracing and detection on infected farms was assessed. The same risk zone classification was used for each scenario.

### The movement control scenario – scenario 2

Animal movement from infected flocks to non-infected areas is crucial in the transmission of Highly Pathogenic Avian Influenza (HPAI). The literature has shown that HPAI transmission is transboundary through various means, including the import of live poultry [67,68]. Hence, movement restriction is considered a disease control and prevention mechanism. Movement control is a strategy to reduce the likelihood of disease spread by reducing the frequency of bird contact. Accordingly, in the movement control scenario simulation, movement restrictions are imposed after 3 days of the HPAI outbreak, with 50% and 90% on days 3 and 7, respectively [69]. The baseline probability parameters for observing clinical signs in HPAI were assigned values of 3, 2, and 1, respectively, for high-risk, moderate-risk, and low-risk zones.

### Intensive Vaccination strategy – scenario 3

Different types of HPAI vaccination exist as a control measure, as noted by both de Leo and Bolzoni [70] and Nickbakhsh et al [28]. HPAI vaccination should only be administered if the action is cost-effective [71]. However, there are insufficient studies on the duration of vaccine-induced immunity, which varies depending on the vaccine formulation and the bird’s overall health. In the laboratory, a single dose of vaccine in chickens at least 3 weeks of age, without maternal antibodies, can provide adequate protection by 3 weeks post-vaccination [72,73]. However, in the field, there are reports of birds being vaccinated multiple times and still becoming clinically affected or dying when exposed to the Avian Influenza virus [74]. In most cases, the vaccine is used either as a preventive measure or an emergency measure. So far, the highest proportion of total vaccines produced has been used by China (90.99%) and Egypt (4.65%) [75].

Despite wider outbreaks of HPAI, countries like the United States of America (USA) have restricted the use of vaccination, mainly due to logistical, political, and economic factors. The USDA explained that the currently available vaccine relies on a two-dose regimen, which can be impractical for distribution to flocks. Another reason is that HPAI is a rapidly evolving virus that requires a vaccine well-matched to the then-circulating strains. Thus, thorough surveillance is essential to track circulating strains of the virus and ensure that virulent strains have not evolved through recombination and genetic assortment. The central economic fact is that many trade agreements do not permit HPAI vaccination due to concerns about importing the virus and poultry products (meat and eggs), as vaccination can only prevent severe disease and death, not infection [71,76]. In other countries, such as those in Europe and the United States, vaccination was not permitted. However, some European countries have recently been permitted to use HPAI vaccines [77].

According to published research on vaccine effectiveness and the commencement of protection in both field and laboratory settings, protection in poultry is not attained before 11–13 days after vaccination [78,79]. All layer chickens tested seronegative again, around 20.5 weeks after receiving two doses of the killed LPAI H6N2 vaccination [6]. In the NAADSM modeling, the authors incorporated a 14-day delay in immunity following vaccination and the implementation of movement restrictions. The vaccine immune period for HPAI-vaccinated poultry was suggested to be 6 months or 180 days [80]. The minimum time that must elapse after the first vaccination for a unit to be queued for the second vaccination was also estimated to be 6–12 months by different studies [81]. This simulation used two intervals: 9 months (270 days). Experts need to repair several diseased units (of any production type) that must be detected before vaccine administration starts. For this modeling purpose, we selected a single farm unit. Furthermore, in the case of HPAIV outbreaks in high-risk areas for farm transmission, emergency protective vaccination is used within a 3-km radius to model the outbreak, based on recommendations [80].

### Depopulation /destruction – scenario 4

Poultry depopulation (stamping out) efficacy was assessed based on the delay in days to depopulate (1 day) and the procedure’s capacity. For this purpose, the number of days (x) and the number of farms that would be depopulated (y) were estimated using relational functional values (x = 1, 2, 3, 7 days; y = 1, 1, 2, 5 farms) as used by Medina et al. [46] along with movement control.

The United Arab Emirates (UAE) employs approved humane depopulation methods to control outbreaks of Highly Pathogenic Avian Influenza (HPAI), prioritizing both rapid disease containment and animal welfare in line with international recommendations. Although specific national guidelines are not always publicly detailed, the UAE, through the Abu Dhabi Agriculture and Food Safety Authority (ADAFSA), follows practices consistent with standards set by organizations such as FAO and USDA-APHIS. Confirmed methods include Ventilation Shutdown (VSD), which reduces airflow in poultry houses to raise temperature and humidity, leading to bird mortality from heat stress and suffocation. This approach is often used when preferred alternatives are not feasible or when a rapid response is required. A variation, Ventilation Shutdown Plus (VSD+), involves the use of carbon dioxide (CO₂) to anesthetize the birds prior to ventilation shutdown, thereby reducing distress and improving animal welfare outcomes [82,83].

### Cost accounting for the various scenarios

Consideration was given to area surveillance expenses for the first scenario, the control scenario, which involves no intervention. On the other hand, zone surveillance and vaccination expenses were considered in the second scenario, which incorporated vaccination as a disease control technique. Zone surveillance expenses were calculated for high-risk, moderate-risk, and low-risk zones. The cost of destruction and depopulation was also monitored in NAADSM modeling for the third scenario, which included zoning and depopulation or destruction as the third disease control mechanism. The costs of destruction used in NAADSM modeling are the cost of appraisal (per unit/farm), costs of cleaning and disinfection (per unit/farm), indemnification (per animal), euthanasia (per animal), and bird disposal (per bird), which are $0.09/farm, $ 997,000/farm, $2/bird, $0.67/bird, and $2.05/bird, respectively. All these costs were estimated based on the survey responses of Seeger et al. [84].

The five categories of destruction costs were assessment costs (per unit or farm), cleaning and disinfection costs (per unit or farm), indemnity costs (per animal), euthanasia costs (per animal), and disposal costs (per bird). These costs were calculated using data from Seeger et al. [84]. Site setup (per unit/farm) and baseline vaccination costs per animal are included in vaccination expenses. In addition to vaccination, the NAADSM requires information on the maximum number of animals that can be vaccinated before costs rise and the additional expense for each vaccinated animal over the threshold (per animal). Expert advice estimated $1,000 in site setup per unit, whereas the literature suggested a baseline vaccine cost of $0.13 per bird [84]. Based on expert advice from our consultation, the estimated costs for zone surveillance in high-risk, moderate-risk, and low-risk zones were $0.03, $0.02, and $0.01, respectively.

### Sensitivity analysis

Model structure optimization and the identification of important model parameters can both benefit from sensitivity analysis (SA) [85]. By altering the detection capability of the performing body for the HPAI virus outbreak in the depopulation scenario and the vaccination capability for the third vaccination scenario, we conducted a sensitivity analysis. Two further levels were chosen for the first parameter (detection). For the initial four scenarios, the detection capacity parameter was maintained at 100, 50, and 25 farms on days 0, 3.5, and 7, respectively. Then, by days 3.5 and 7 for scenario 1 of the sensitivity study, the detection capability had changed by 40% and 100%, respectively. Similarly, by days 3.5 and 7 for scenario 2 of the sensitivity analysis, it had changed by 40% and 180%, respectively. Likewise, at an additional level, vaccination capacity increased by 100%, 33%, and 0% on days 1, 4, and 7 following vaccination, respectively (Table 4). In addition, in the vaccination sensitivity analysis, the minimum time that must elapse after the first vaccination before a unit could be queued for a second vaccination was increased from the baseline 9 months (270 days) to 360 days when modeling the single-dose recombinant vaccination option [86,87].

**Table 4:** Parameter levels for sensitivity analysis.

	Detection capacity				Vaccination capacity	
Days	Base capacity	Level 1	Level 2	Days	Base capacity	Level −1
**0**	100	100	100	0	0	0
**3.5**	50	70 (40%)	70 (40%)	1	5	10 (100%)
**7**	25	50 (100%)	70 (40%)	4	15	20 (33%)
				7	35	35 (0)

### Results and discussion

The results of the NAADSM simulation for each scenario—Scenario 1 (no intervention), Scenario 2 (movement control), Scenario 3 (intensive vaccination), and Scenario 4 (depopulation) are discussed hereafter, following the discussion of infection exposure of poultry birds.

### Poultry farms’ exposure to the HPAI

The simulation results show that indirect contact spread has the most significant effect in adequately exposing birds to HPAI. Compared with direct contact, indirect contact exposure is higher than direct contact spread in all scenarios, indicating the importance of airborne spread as a primary HPAI transmission mechanism. The total number of birds directly exposed to the infected heads of birds for scenarios 1, 2, 3, and 4 is 872, 852, 862, and 881 thousand, while the number of birds indirectly exposed to infection is 4 million for scenarios 1 and 4; and 5 million for scenarios 2 and 3 (Table 5). The exposure of poultry birds to infection increases by 357%, 442%, 431%, and 376% for indirect contact transmission mechanisms compared to direct mechanisms in scenarios 1, 2, 3, and 4, respectively. In general, the number of birds exposed to infected birds, either directly or indirectly, is 12 in scenarios 1 and 2, 15 in scenario 3, and 13 in scenario 4. This implies that no action can reduce the birds’ exposure probability to infected entities, either directly or indirectly.

**Table 5:** Poultry farms and birds’ exposure to infection.

	Scenario 1		Scenario 2		Scenario 3		Scenario 4	
Output description	Mean number of birds (SD)	Mean farm number (SD)	Mean number of birds (SD)	Mean farm number (SD)	Mean number of birds (SD)	Mean farm number (SD)	Mean number of birds (SD)	Mean farm number (SD)
**Number of units adequately exposed by direct contact**	872,316 (1,879,813)	2 (1.43)	851,856 (1,818,098)	2 (1.43)	861,676 (1,534,828)	2 (1.41)	881,390 (1,800,766)	2 (1.00)
**Number of units adequately exposed by indirect contact**	3,988,316 (3,837,378)	10 (6.00)	4,616,109 (4,090,421)	11 (6.75)	4,575,408 (3,857,645)	11 (7.00)	4,193,970 (4,155,803)	10 (7.58)
**Total number of units adequately exposed by direct contact with an infected unit**	336,996 (867,075)	1 (1)	360,501 (797,239)	1 (1.12)	439,263 (1,199,107)	1 (1.12)	304,333 (681,436)	1 (1.00)
**Total number of units indirectly exposed to an infected unit**	12,344,066 (8,324,025)	31 (17.00)	14,148,866 (9,412,511)	33 (18.51)	14,290,086 (9,261,954)	34 (18.74)	12,733,294 (10,376,383)	31 (21.41)
**Number of units exposed by any contact (direct/ indirect) to any infected unit**	12,681,062 (8,475,292)	32 (18.00)	14,509,367 (9,557,110)	34 (18.99)	14,729,349 (9,485,089)	35 (19.24)	13,037,627 (10,547,520)	31 (21.95)

SD: Standard Deviation.

### Baseline/no intervention scenario-1 results

**Fig 2:**
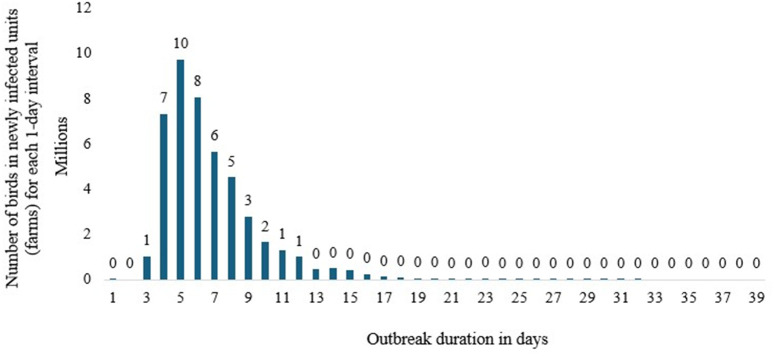
The Epidemic curve output for scenario 1 (baseline/no-intervention scenario).

The result, depicted in Fig 2, shows that the disease outbreak can continue for approximately 31 days in scenario 1 (baseline/no intervention), with the number of poultry birds detected each day reaching a peak of around 10 million on the fourth day of the outbreak. A prolonged disease outbreak may increase the likelihood of human infection and other related negative economic impacts, such as disruptions to international trade and commerce. The results also showed that the total number of poultry birds susceptible to disease or HPAI infection is approximately 45 million, while the number of latent birds is 5 million (Table 6). Similarly, the number of birds that are or become clinically overt, causing observable and recognizable symptoms, and those that are subclinical, not detectable, or producing effects not detectable by usual clinical tests, is 45 million. The number of poultry birds infected by the virus in this baseline scenario is 45 million. The mean duration of the disease outbreak and the active disease phase in the designated iteration are approximately 26 days in this scenario (Table 6). Most poultry birds (44 million) are detected for their HPAI infection by exhibiting clinical signs, while only about 1.7 million birds are identified through diagnostic testing (Table 6).

**Table 6:** Simulation results for the four scenarios (baseline, movement control, vaccination, and depopulation).

	Scenario 1		Scenario 2		Scenario 3		Scenario 4	
Output description	Mean number of birds (SD)	Mean farm number (SD)	Mean number of birds (SD)	Mean farm number (SD)	Mean number of birds (SD)	Mean farm number (SD)	Mean number of birds (SD)	Mean farm number (SD)
**The total number that becomes susceptible**	51,317,848 (1,228,627)	45 (0.00)	51,331,597 (1,206,679)	45 (0.40)	51,368,923 (1,273,667)	45 (0.41)	51,159,821 (7,049)	45 (0.04)
**The total number that becomes latent**	4,881,246 (6,081,782)	5 (2.00)	4,894,681 (6,134,020)	5 (2.44)	5,321,000 (6,450,941)	5 (2.27)	4,235,918 (6,030,260)	5 (2.75)
**The total number that becomes subclinical**	45,200,834 (16,574,552)	40 (14.00)	45,414,679 (16,419,781)	40 (14.12)	45,525,502 (16,297,345)	40 (14.00)	37,800,726 (22,412,800)	33 (19.02)
**The total number that becomes clinical**	45,200,834 (16,574,552)	40 (14.00)	45,414,679 (16,419,781)	40 (14.12)	45,525,502 (16,297,345)	40 (14.00)	36,613,183 (21,745,761)	30 (17.58)
**The total number that becomes naturally immune**	4,799,362 (6,259,903)	4 (2.00)	4,658,981 (60,33,148)	4 (2.40)	4,431,045 (5,736,085)	4 (2.31)	232,679 (1,371,982)	0.12 (0.36)
**Number vaccinated for any reason**	0	0	0	0	43,957,502 (15,946,018)	40 (13.14)	0	0
**Becomes vaccine-immune**	0	0	0	0	2,316 (5,418)	0.21 (0.44)	0	0
**The total number that is destroyed**	0	0	0	0	0	0	37,549,311 (22,268,190)	33(18.77)
**The total number of dead in all units from disease**	40,401,472 (15,919,996)	36 (13.00)	40,755,699 (15,753,693)	36 (12.82)	41,094,456 (15,611,139)	36 (12.75)	1,711,599 (3,646,846)	1 (1.14)
**Number in all units that become infected (not including initially infected)**	45,191,834 (16,574,552)	39 (14.00)	45,405,679 (16,419,781)	39 (14.12)	51,393,281 (45,516,502)	39 (14.00)	37,792,218 (22,413,091)	32 (19.03)
**Total number of units identified successfully by tracing (forward/ back) of direct contact**	707,857 (2,105,491)	1 (1.00)	707,907 (1,859,100)	1 (1.12)	769,545 (2,083,676)	1 (1.12)	505,965 (1,407,492)	1 (1.02)
**Number of units identified by tracing (forward or backward) of indirect contact**	21,559,057 (15,595,713)	31 (17.00)	22,067,186 (15,930,168)	31 (18.46)	23,087,993 (16,239,162)	34 (18.69)	19,779,959 (17,318,871)	29 (20.88)
**Successfully identified by tracing (forward/back) of contact (direct/indirect)**	22,266,914 (16,038,239)	31 (18)	22,775,093 (16,193,761)	34 (18.93)	23,857,538 (16,680,992)	35 (19.20)	20,285,925 (17,677,457)	30 (21.42)
**Total number in all units vaccinated for any reason**	0	0	0	0	43,957,502 (15,946,018)	40 (13.14)	0	0
**Number of units detected by clinical signs**	44,095,134 (16,366,911)	39 (14.00)	4,419,7223 (16,208,307)	39 (13.89)	44,451,685 (16,127,992)	40 (13.81)	35,665,323 (21,333,607)	30 (17.28)
**Number of units detected by diagnostic testing**	1,650,786 (3,023,478)	3 (2.00)	2,777,806 (3,165,754)	5 (3.13)	2,729,524 (2,767,907)	5 (3.19)	2,621,924 (3,413,323)	5 (3.48)
**Duration of the active disease phase**	26 (7.00)	26 (7.00)	32 (8.89)	32 (8.89)	31 (8.63)	31 (8.63)	15 (5.97)	15 (5.97)
**Duration of the outbreak**	26 (7.00)	26 (7.00)	32 (8.82)	32 (8.82)	31 (8.55)	31 (8.55)	16 (6.17)	17 (6.17)

SD: Standard Deviation.

### Movement control scenario-2 results

**Fig 3:**
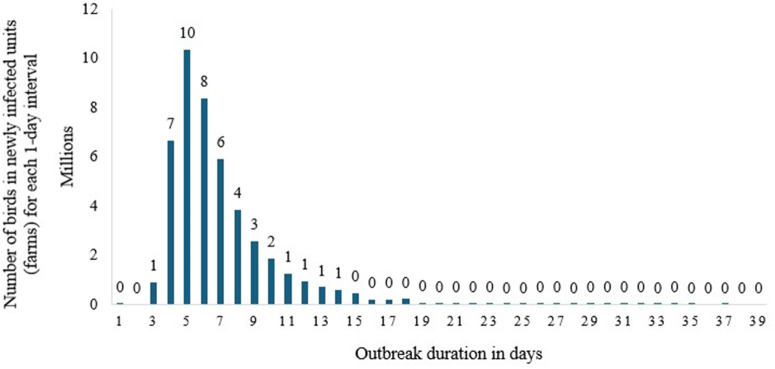
The Epidemic curve output for scenario 2 (Movement control).

The epidemic curve output for Scenario 2, Fig 3, indicates that the disease can persist for 36 days once initiated. However, both the active disease duration and the disease outbreak duration are 32 days (Table 6). The outbreak peaked at approximately 10 million birds on the fourth day after the initial outbreak. Fig. In this scenario, the total susceptible poultry population is 51, while the subclinical and clinical bird populations are 45 million each. Similarly, the latent population is 6 million (Table 6). Of the 45 million birds infected with HPAI, approximately 91% (4 million) die, while only 9% (4 million) develop natural immunity, implying that most birds die.

### Intensive vaccination scenario-3 results

**Fig 4:**
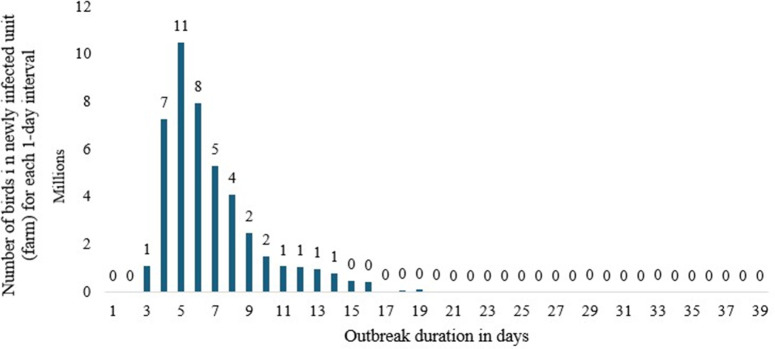
The Epidemic curve output for scenario 3 (intensive vaccination).

The result under the third scenario (Fig 4) indicates that the disease peak outbreak will occur on the fourth day, resulting in the death of approximately 11 million poultry birds. The outbreak may last up to 49 days in scenario two, which involves intensive vaccination. The total number of poultry birds that become susceptible to disease is approximately 51 million, while the number of latent poultry birds is around 5 million. The result further shows that the total number of poultry birds that will become subclinical and clinical is 46 million heads each. The number of poultry birds that become naturally immune is 5 million, while only around two thousand poultry birds become vaccine immune. During the outbreak, approximately 44 million poultry birds were vaccinated, while around 51 million were HPAI-infected, out of which 41 million died due to infection. This shows that most disease-infected birds (80%) die. Backwards and forward tracing are important in identifying and mapping disease routes in the infection area. In this simulation, approximately 24 million birds would be tracked using direct or indirect forward- and backward-tracing methods (Table 6).

### Depopulation/destruction- scenario 4

**Fig 5:**
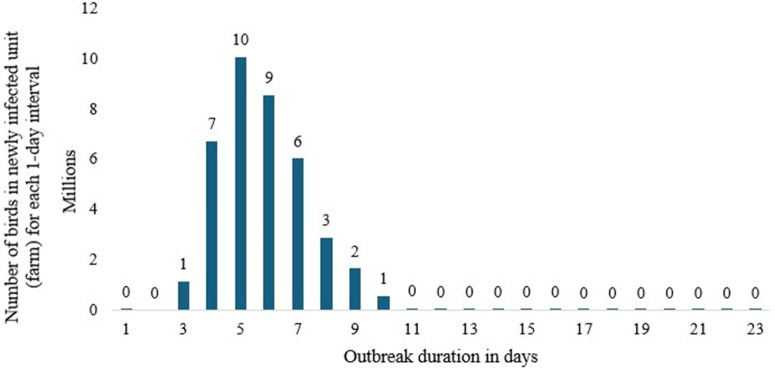
The Epidemic curve output for scenario 4 (depopulation).

The results show that disease transmission peaks 4 days after onset, as in other cases. At the same time, about 10 million poultry birds were discovered per day on the fourth day of the outbreak (Fig 5). In contrast to scenarios 1, 2, and 3, which are nearly double of this scenario, the simulation result for scenario 4 (depopulation or destruction of poultry) shows that the duration of the active disease phase would be shortened to fifteen days (with a maximum of 36 days and a minimum of 5 days). The disease outbreak duration would average 16 days, ranging from 5 to 37 days (Table 6). This finding aligns with FAO (2011) and Stegeman et al (2004) [88,89], which reported that depopulation or culling helps reduce the risk of disease transmission in the surrounding area and is commonly implemented during outbreaks on broiler farms. According to this study, 51 million poultry birds would become sensitive in this scenario.

On the other side, there were around 4 million latent birds (Table 6). The number of naturally immune birds would be nearly halved in scenario 4, indicating that natural immunity is much lower than in the other three scenarios (scenarios 1, 2, and 3). Approximately 20 million (53%) of the 38 million depopulated birds were destroyed or depopulated in the depopulation scenario after being discovered infected with the HPAI virus, using various tracking methods (forward and backward tracing). Furthermore, the findings indicate that 38.51 million susceptible birds would contract the virus, and virtually all (more than 99%) infected birds would be killed during the subsequent outbreak.

There is a significant difference between depopulation and the other three scenarios in the duration of the disease outbreak and the active disease period. Depopulation would significantly reduce the duration of the outbreak and the active disease phase. This result is consistent with other findings, which report the same outcome: that depopulation is more effective in reducing the number of infected flocks and the duration of the epidemic [90–95]. However, there is a strong argument that the effectiveness of depopulation comes at the cost of depopulation of more flocks, which is associated with higher costs. Hence, the selected scenario should be evaluated based on the disease control measures’ objective (i.e., financial cost reduction or epidemic size) [96]. Hence, increasing biosecurity and reducing the first infection rate are important HPAI control mechanisms [66,97].

The results across all the above-discussed scenarios show no significant difference in the number of susceptible or latent birds to the disease. Similarly, the number of birds that are or become clinically overt (causing observable and recognisable symptoms) and sub-clinical (not detectable or producing effects not detectable by usual clinical tests) is almost the same across all scenarios. The number of birds that become naturally immune is higher in the first, second, and third scenarios than in the fourth (depopulation) scenario. In contrast, only an insignificant poultry bird becomes vaccine-immune in scenario 4, while there is no naturally immune poultry in the other three scenarios.

Literature affirmed that vaccines have reduced a flock’s susceptibility to HPAI infection, reduced the quantity of virus shed post-challenge, reduced transmission, and markedly reduced disease losses [94]. However, this research finding revealed that vaccination during the outbreak does not reduce the number of infected poultry birds, as the number of infected units is almost the same across all three scenarios (1–3). There is also no significant difference between the number of poultry that died of HPAI disease among the three (1st, 2nd, and 3rd) scenarios. However, this figure is reduced by approximately 95% in scenario 4. Depopulation may include depopulating infectious farms, preemptively depopulating contiguous premises, banning restocking on emptied farms, and reducing the number of contacts among farms, effectively reducing virus transmission over time.

Both scenarios 2 (movement control) and 3 (vaccination) have prolonged active disease phases and outbreak durations. This finding aligns with others’ arguments that vaccination campaigns can incur additional expenses by prolonging epidemics or disrupting global trade. However, vaccination can be the approach linked to the fewest depopulated flocks or birds, potentially lowering the costs of an outbreak. [95]. Meanwhile, for the fourth scenario, depopulation or destruction, the duration of the active disease phase was reduced by 53% and 52% compared to the movement control and vaccination scenarios, respectively. The disease outbreak durations decreased by 50% and 48% compared to scenarios 1 and 2.

Poultry farms’ direct and indirect contact is another intriguing finding in this study. The findings showed that, among all the poultry birds traced, a notably greater number were traced through indirect contact; nearly 95% were traced through indirect contact (Table 5). This result is supported by the authors’ claim that direct bird contact rates among commercial poultry farms are negligible [46]. However, the 5% trace of direct contact is not insignificant and needs to be considered when developing HPAI control policies and intervention plans. In general, it is also apparent from the results that indirect exposure to infected units plays the most significant role in the transmission of HPAI disease. On the other hand, scenarios 2 (movement control) and 3 (vaccination) would increase birds’ exposure to HPAI through any of the means of spread (direct contact, indirect contact, airborne spread, and local area spread). This finding is also consistent with Bauzile et al. [98], who realized that the transit of transport vehicles created more opportunities for transmission than the exchange of live animals or direct animal contact.

### Cost accounting for the control strategies scenarios

In any intervention, the cost of implementation should be given due attention across financial, economic, and social aspects. Any disease outbreak in a given country will have all these costs to prevent and restore the situation after the outbreak. HPAI virus outbreak control in the two scenarios revealed a significant difference in financial costs between vaccination and depopulation (destruction).

The term “surveillance cost” refers to the direct and indirect expenses associated with identifying, tracking, and monitoring the presence and spread of the disease in chicken populations. To prevent major epidemics, surveillance is crucial for early detection and prompt management. The major direct costs include sampling and testing expenses, staff costs (including veterinary wages and allowances), logistics and equipment costs, and program design and administration costs. The opportunity cost of labor and farmers’ time, as well as the expenses resulting from market disruption, are examples of indirect costs. This model includes only direct cost components. Accordingly, the simulation output (results) of surveillance and monitoring costs for each scenario is presented in Table 7. The results show that scenarios 2 and 3 have the highest surveillance costs, at approximately $34.4 million. The least expensive option for monitoring is scenario 4, at about USD 2 million, followed by scenario 1.

**Table 7:** Surveillance costs for scenarios 1, 2, 3, and 4.

Variable description	Scenario-1	Scenario-2	Scenario-3	Scenario-4
**Unit days in zone**	831 (318)	1049 (396)	1044 (388)	115 (61)
**Animal days in the zone**	919,966,615 (369,316,656)	1,146,247,824 (475,003,310)	1,151,549,864 (450,239,356)	71,206,851 (56,132,459)
**Cost of surveillance**	27,377,557 (11,004,087)	34,443,063 (13,393,073)	34,416,189 (13,258,960)	1,971,766 (1,593,039)

SD: Standard Deviation.

The results indicate that the cost of depopulation would be approximately 36 times higher than vaccination (Table 8). Costs of depopulated birds’ disposal for the birds destroyed and indemnification (compensation for re-stocking of the poultry farms) composed the major share of the cost of depopulation, each accounting for 36.65% and 35.76% of the total cost, respectively, followed by the total cost of cleaning and disinfection for the whole units destroyed (Table 8).

**Table 8:** Cost accounting for scenarios 3 and 4 (USD).

Cost Item Description	Mean for Scenario 3 vaccination(SD)	Mean for scenario 4 depopulation (SD)
**Total cost of appraisal for all depopulated units**	n/a	133,075 (75,258)
**Total cost of cleaning and disinfection for all units destroyed**	n/a	32,757,882 (18,525,660)
**The total cost of euthanasia for all depopulated birds**	n/a	24,123,364 (14,488,645)
**Total cost of indemnification for all birds in depopulated units**	n/a	73,885,042 (44,026,238)
**Total cost of depopulated bird disposal for all birds**	n/a	75,732,168 (45,126,894)
**The total cost associated with depopulation**	0	206,631,531 (122,044,710)
**Total cost of vaccination setup**	39,785 (13,138)	n/a
**Total cost of vaccination for all units vaccinated**	5,714,475 (2,072,982)	n/a
**Total cost associated with vaccination**	5,754,260 (2,085,931)	0
**The total cost of all activities over the cost of the iteration**	5,754,260 (2085931)	206,631,531 (122,044,710)

SD: Standard Deviation.

### Sensitivity analysis

For the sensitivity analysis, two levers were tested: small changes to vaccination parameters (capacity and a recombinant‑vaccine specification) and increases in detection capacity under the depopulation strategy. Results are summarized in Table 9. Adjusting vaccination throughput or switching to a recombinant vaccine produced only small changes. With higher capacity, direct contact infections declined approximately 16%, whereas under the recombinant vaccine specification, they rose 9%. Immune-related outputs moved slightly: birds becoming vaccine‑immune fell by 1–3%, and naturally immune birds dipped by 3%; the number vaccinated changed by 1%. Crucially, outbreak timing did not change (no shortening of the active phase or total duration). In short, within the tested ranges, vaccination adjustments during an active outbreak had a limited epidemiological effect. Raising detection capacity had much larger consequences: disease-related deaths fell substantially (by ~40%), culling increased, naturally immune birds declined (consistent with earlier removal), and detections shifted toward earlier clinical recognition; indirect-contact transmission also decreased. As with vaccination, the timing of outbreaks remained stable.

**Table 9:** Sensitivity analysis for detection and vaccination capacity changes.

Epidemiological outputs	Vaccination baseline scenario	vaccination capacity increased	vaccination – Recombinant Vaccines	Sensitivity to vaccine capacity (%change)	Sensitivity to Recombinant Vaccines (%change)	depopulation baseline scenario	Detection capacity increased-1	Detection capacity increased-2	Sensitivity to detection capacity-1 (%change)	Sensitivity to detection capacity-2 (%change)
**Total number of birds infected by direct contact**	439,263	367,080	478,912	−16%	9%	304,333	351,358	325,739	15%	7%
**Total number of birds infected by indirect contact**	4,575,408	4,643,520	4,548,196	1%	−1%	4,193,970	4,034,387	3,809,098	−4%	−9%
**The total number that becomes susceptible**	51,368,923	51,312,114	51,378,164	0%	0%	51,159,822	51,159,575	51,159,575	0%	0%
**Total number of birds that become latent**	5,321,000	4,847,191	5,512,605	−9%	4%	4,235,918	4,195,740	4,322,143	−1%	2%
**Total number of birds that become subclinical**	45,525,502	45,285,730	45,443,818	−1%	0%	37,800,726	37,639,918	38,397,922	0%	2%
**Total number of birds that become clinical**	45,525,502	45,285,730	45,443,818	−1%	0%	36,613,183	36,421,362	37,171,309	−1%	2%
**The total number of birds that become naturally immune**	4,431,045	4,309,379	4,424,213	−3%	0%	232,679	174,097	90,398	−25%	−61%
**Epidemiological outputs**	**Vaccination-based scenario**	**vaccination capacity increased**	**vaccination - Recombinant Vaccines**	**Sensitivity to vaccine capacity (%change)**	**Sensitivity to Recombinant Vaccines (%change)**	**Based scenario-depopulation**	**Detection capacity increased-1**	**Detection capacity increased-2**	**Sensitivity to detection capacity-1 (%change)**	**Sensitivity to detection capacity-2 (%change)**
**Number vaccinated for any reason**	43,957,502	43,720,633	43,869,353	−1%	0%	0	0	0	0%	0%
**Becomes vaccine-immune**	2,316	2,290	2,250	−1%	−3%	0	0	0	0%	0%
**Total number that are destroyed**	0	0	0	0	0	34,774,249	35,526,386	36,336,851	2%	4%
**Total number of dead in all units from disease**	41,094,456	40,976,351	41,019,605	0%	0%	1,711,599	1,014,733	1,019,313	−41%	−40%
**Number of birds detected by clinical signs**	44,451,685	44,097,101	44,388,378	−1%	0%	35,665,323	36,263,073	37,067,329	2%	4%
**Number of birds detected by diagnostic testing**	2,729,524	2,579,139	2,786,084	−6%	2%	2,621,924	2,406,462	2,389,492	−8%	−9%
**Duration of the active disease phase**	31	31	31	0%	0%	15	15	15	0%	0%
**Duration of the outbreak**	31	31	31	0%	0%	16	16	16	0%	0%

NAADSM treats each farm as a homogeneous unit with fixed contact rates; it does not explicitly capture company‑level networks, dynamic rerouting, or behavioural/compliance effects, and it excludes backyard/wild interfaces by design. Parameters and costs are based on the literature and expert inputs, and our sample covers 46 commercial operations. Hence, the results are directional and sector-specific rather than point forecasts for the entire system. These structural features limit generalisation beyond the modelled network.

## Conclusions and recommendations

This study was conducted with the objectives of simulating the spread of Highly Pathogenic Avian Influenza (HPAI) virus using a UAE-customized animal disease spread model to analyze prevalence based on production types, identify the implications of HPAI control strategies, and estimate associated government costs. Furthermore, the study aimed to understand the presence, transmission, and prevalence of the HPAI virus to inform better and more effective intervention measures alongside disease control measures. The data pertinent to poultry farm information was obtained from the Abu Dhabi Agriculture and Food Safety Authority (ADAFSA) and the Ministry of Climate Change and Environment (MOCCAE) in the United Arab Emirates (UAE). Model parameters were collected from published and unpublished materials. Four scenarios (no intervention, movement control, vaccination, and depopulation/destruction) were modeled and evaluated.

The fourth scenario, which involves the depopulation or destruction of poultry birds, has been found to drastically shorten the duration and other consequences of HPAI disease spread. Hence, the UAE is keen to consider the optimal control strategy to enhance biosecurity and preparedness against HPAI outbreaks at poultry farms. It is essential to be aware of the government’s cost for each control strategy to allocate government funds accordingly. Therefore, the choice of scenarios for implementation must be carefully considered from various perspectives. In other words, HPAI is a highly contagious infectious disease that economically impacts poultry farms and, ultimately, consumers. Therefore, even at considerable expense, the government may adopt drastic measures such as depopulating diseased birds and compensating farmers for their losses. In general, if policymakers decide to vaccinate, preventive vaccination is the optimal strategy to minimize the duration of the disease outbreak. Booster vaccinations should be conducted in the most susceptible and infectious poultry species in high-risk transmission areas.

The United Arab Emirates and other nations in the Gulf region and the Eastern Mediterranean Region have identified avian influenza as one of high priority animal diseases and the most significant zoonotic illnesses at present, thus leading government authorities to work together with the universities to improve the insight prospective approach of preparedness, contingency planning and simulation approach of evaluation.

The main aim of the HPAI scenario analysis in this study is to enable policy and decision-makers to make informed choices to enhance the country’s preparedness against HPAI outbreaks and their social and economic implications for the poultry sector. For example, to successfully prevent and control HPAI, a regional, multidisciplinary, and coordinated One Health approach is required to strengthen surveillance and monitoring systems, early warning systems, risk assessments, and the timely dissemination of information to public health authorities. Furthermore, a comprehensive study is necessary to understand the epidemiology and transmission dynamics in the country and to implement real-time surveillance to track circulating strains of the virus, ensuring that virulent strains have not evolved due to recombination and genetic assortment. Also, sound vaccination monitoring systems and evidence-based knowledge are needed to ensure the effectiveness of vaccination programs. In general, depopulation has become the most effective scenario for controlling HPAI outbreaks. Hence, policymakers, development practitioners, and farm owners shall work jointly to select the most suitable scenario options that minimize the overall negative impacts of an outbreak.

## Supporting information

S1 File See Supplemental information for sensitivity analysis. (XLSX)
